# Dihydromyricetin Alleviates Pulmonary Fibrosis by Regulating Abnormal Fibroblasts Through the STAT3/p-STAT3/GLUT1 Signaling Pathway

**DOI:** 10.3389/fphar.2022.834604

**Published:** 2022-03-14

**Authors:** Zhen Li, Jing Geng, Bingbing Xie, Jiarui He, Jing Wang, Liang Peng, Yinan Hu, Huaping Dai, Chen Wang

**Affiliations:** ^1^ Department of Pulmonary and Critical Care Medicine, Center of Respiratory Medicine, China-Japan Friendship Hospital; National Center for Respiratory Medicine; National Clinical Research Center for Respiratory Diseases; Institute of Respiratory Medicine, Chinese Academy of Medical Sciences, Peking Union Medical College, Beijing, China; ^2^ State Key Laboratory of Medical Molecular Biology, Department of Physiology, Institute of Basic Medical Sciences Chinese Academy of Medical Sciences, School of Basic Medicine Peking Union Medical College, Beijing, China; ^3^ Institute of Clinical Medical Sciences, China-Japan Friendship Hospital, Beijing, China

**Keywords:** idiopathic pulmonary fibrosis, dihydromyricetin, fibroblast, myofibroblast, GLUT1, glucose metabolism

## Abstract

**Background:** Idiopathic pulmonary fibrosis (IPF) is a chronic and progressive disorder with a poor prognosis. Although dihydromyricetin (DHM), extracted from vine tea and other Ampelopsis species, has been proven to have anti-inflammatory and antioxidant functions, the effects of DHM on IPF remain unclear.

**Methods:** The effects of DHM on the differentiation, migration, proliferation, and respiratory functions of primary mouse lung fibroblasts (PMLFs) and primary human lung fibroblasts (PHLFs) were detected by western blotting, the Transwell assay, EdU staining, and the Mito Stress test. Then, the impacts of DHM on bleomycin (BLM)-induced pulmonary fibrosis were evaluated by pathological staining, western blotting, and coimmunofluorescence staining. The signaling pathway influenced by DHM was also investigated.

**Results:** DHM could regulate the differentiation of fibroblasts to myofibroblasts and suppress the abnormal migration, proliferation, and respiratory functions of myofibroblasts induced by TGF-*β*1 or myofibroblasts from IPF patients. DHM could also alleviate pulmonary fibrosis induced by BLM. All these effects were achieved by regulating the STAT3/p-STAT3/GLUT1 signaling pathway.

**Conclusion:** DHM could regulate the abnormal functions of myofibroblasts induced by TGF-*β*1 and myofibroblasts from IPF patients and alleviate pulmonary fibrosis induced by BLM; thus, DHM might be a candidate medicinal treatment for IPF.

## Introduction

Idiopathic pulmonary fibrosis (IPF), characterized by chronic and progressive fibrosis of the interstitial lung tissue, is an irreversible and fatal lung disease with a poor prognosis (Richeldi et al., 2017). Historical clinical evidence suggests that the median survival of untreated IPF patients is approximately 2–3 years after diagnosis, and the mortality rate of IPF patients remains high, although some antifibrotic treatments (pirfenidone and nintedanib) have been adopted (Lederer and Martinez, 2018). Therefore, new drugs for the treatment of IPF patients are urgently needed.

The histopathological characteristics of IPF are the formation of fibroblast foci and deposition of disordered collagen and an extracellular matrix (ECM) (Wolters et al., 2014). Although the precise initiation factors that lead to these disorders remain unknown, it is widely recognized that the pathological process of IPF is related to fibroblasts and myofibroblasts (Chanda et al., 2019). Previous studies have suggested that various cytokines, such as the transform growth factor (TGF-*β*), and signaling pathways, such as the STAT/pSTAT3 pathway, can promote fibroblast differentiation into myofibroblasts and ultimately result in fibrosis of the lung tissue (Zehender et al., 2018). For this reason, restraining the differentiation of fibroblasts into myofibroblasts may block the initiation of fibrosis, while reversing this differentiation process might lead to a breakthrough treatment for IPF (Wei et al., 2019).

**FIGURE 1:**
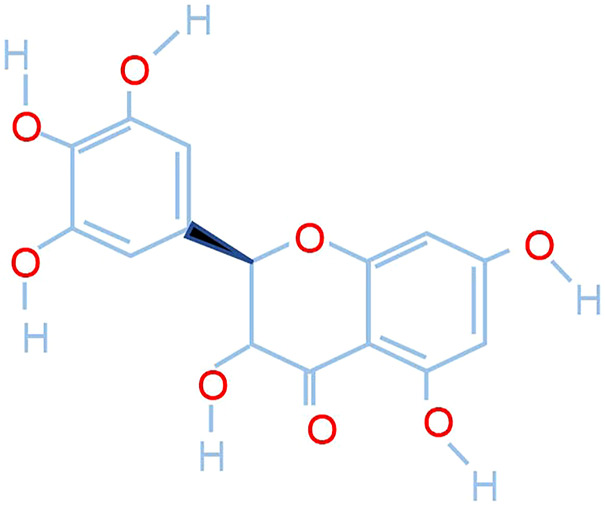
Chemical structure of dihydromyricetin.

Dihydromyricetin (DHM, the structure of which is shown in Figure 1), a natural flavonoid extracted from vine tea and other Ampelopsis species, has anti-inflammatory and antioxidant effects and has been proven to inhibit liver fibrosis and diabetic nephropathy (Liu et al., 2019; Zhou et al., 2021). However, its effects on pulmonary fibrosis have not been determined. As inflammation and oxidative stress participate in the process of lung fibrosis (Heukels et al., 2019; Otoupalova et al., 2020), we hypothesized that DHM would be a good potential drug for the prevention and treatment of pulmonary fibrosis.

To test the feasibility of our hypothesis, we detected the effects of DHM on the differentiation, proliferation, and migration of primary fibroblasts from the lungs of IPF patients and control subjects or those from mice, and to explore whether the STAT3/p-STAT3/GLUT1 signaling pathway is involved in these effects, we also used bleomycin (BLM) to induce pulmonary fibrosis in male mice and confirmed the effects of DHM in the mouse model. Our findings suggest that DHM could alleviate pulmonary fibrosis in the mouse model and restrain the differentiation, proliferation, and migration of fibroblasts or myofibroblasts *via* the STAT3/pSTAT3/GLUT1 signaling pathways. Together, our studies suggest that DHM may be a novel treatment for IPF patients.

## Materials and Methods

### Human Samples

Lung tissues were collected from IPF patients (*n* = 9) and donors (*n* = 6) during lung transplantation surgery. The diagnosis of IPF was made according to consensus diagnostic criteria from the American Thoracic Society (ATS)/European Respiratory Society (ERS) (Raghu et al., 2019). All patients provided informed written consent. The study was approved by the Human Assurance Committee of the China–Japan Friendship Hospital.

### Animals

WT C57BL/6 male mice aged 8–10 weeks were purchased from GemPharmatech Co., Ltd. (Nanjing, China). All animals were bred in a specific pathogen-free environment at the China–Japan Friendship Hospital and randomly assigned to four groups: 1) the saline group, 2) the DHM group, 3) the BLM (Hanhui Pharmaceuticals Co., Ltd.) group, and 4) the BLM + DHM group. Pulmonary fibrosis was induced by BLM treatment. The mice in the BLM group and the BLM + DHM group were anesthetized with 1% pentobarbital sodium (50 mg/kg) and intratracheally administered 1.5 U/kg BLM in 50 μL of sterile saline. For mice in the saline group and the DHM group, BLM was replaced with the same volume of saline. DHM was administered by gavage to mice in the DHM group and the BLM + DHM group on day 14 after BLM administration and every day until day 27. Finally, the mice were euthanized on day 28 to analyze pulmonary fibrosis. All experimental procedures complied with the International Association of Veterinary Editors guidelines of 2010 and the Guide for the Care and Use of Laboratory Animals, 8th edition, 2011. All protocols were approved by the Institutional Animal Care and Use Committee of the China–Japan Friendship Hospital (zryhyy11-20-09-4).

### Cell Culture

Primary mouse lung fibroblasts (PMLFs) were isolated from the lung tissue from wild-type (WT) C57BL/6 male mice at 2 weeks of age. Primary human lung fibroblasts (PHLFs) were isolated from the fibrotic lung tissue of IPF patients (IPF-HLF) or the normal lung tissue of donors (N-HLF) (Wang Q. et al., 2021). All cells were verified by coimmunofluorescence analysis with anti-fsp-1 (Proteintech, 66489-1-Ig, 1:200) and anti-alpha-smooth muscle actin (*α*-SMA) (Abcam, ab124964, 1:200). The purity of the cells used in our study was more than 90%. All the cells were cultured in Dulbecco’s modified Eagle’s medium (DMEM) containing 20% fetal bovine serum (FBS) and penicillin/streptomycin at 37°C and 5% CO_2_ and used between passages 3 and 5. PMLFs were stimulated with DHM (100 μM/200 μM/300 μM) (Ruifensi, Chengdu, China) or an equal amount of DMSO (Sigma, C6164) 1 h before treatment with mouse recombinant TGF-*β*1 (15 ng/ml) (MCE, HEK293) for 36 h, and IPF-HLFs and N-HLFs were treated with only DHM (100 μM/200 μM/300 μM) or an equal amount of DMSO for 36 h.

### Cell Viability Assay

The viability of PMLFs and PHLFs was detected by the Cell Counting Kit-8 (CCK-8) assay (Beyotime, Shanghai, China) (Wang et al., 2020). Initially, 5 × 10^3^ cells/well were seeded in 96-well plates and then treated with different concentrations of DHM (100 μM, 200 μM, 300 μM, 400 μM, or 500 μM) or 1‰ DMSO for 36 h. After the treatment was finished, 5 mg/ml CCK-8 reagent was added and incubated for 2 h, and cell viability was quantified by calorimetry.

### Western Blotting and Antibodies

Lung tissues or fibroblasts were homogenized in RIPA lysis buffer (Beyotime, Shanghai, China), to which two kinds of protease inhibitor cocktails (MCE, HY-K0022) had also been added. The proteins were separated on polyacrylamide gels and finally detected with a chemiluminescence substrate system (Bio–Rad Laboratories, CA, United States and Tanon, Shanghai, China) (Miao et al., 2020). The antibodies used for western blotting were as follows (Table 1).

**TABLE 1:** Information of the primary antibody used for cells and model mice.

Primary antibody	For cells	For model mice
Anti-*β*-ACTIN	Proteintech, 66009-1-Ig, 1:5000 42kd	
Anti-fibronectin	Proteintech,15613-1-AP, 1:1000 270kd	Abcam, ab2413, 1:1000 280kd
Anti-Col1a1	Proteintech, 66761-1-Ig, 1:1000 130kd	CST, 81375 1:1000 220kd
Anti-*α*-SMA	Proteintech, 14395-1-AP, 1:1000 42kd	
Anti-GLUT1	Proteintech, 66290-1-Ig, 1:1000 54kd	
Anti-STAT3	CST, 9139, 1:1000 86kd	Proteintech, 60199-1-Ig, 1:1000 88kd
Anti-p-STAT3	CST, 9145, 1:1000 86kd	

### Cell Migration Assay

Cell migration was detected by using Transwell inserts with membranes with an 8.0 μM pore size (Corning, MA, United States) according to previously reported methods. A total of 3 × 10^4^ cells were suspended in 2% FBS containing DMEM and seeded into the upper chambers. DMEM containing 20% FBS was added to the lower chambers as a chemoattractant for cells in the upper chambers. After incubation for 24 h, cells that had migrated through the membrane were stained with 1% crystal violet (Solarbio, Beijing, China).

### Cell Proliferation Assay

PMLFs and PHLFs were cultured in 96-well plates at a density of 5 × 10^3^ cells/well. Cell proliferation was detected by using the EdU proliferation assay (RiboBio, Guangzhou, China). After being seeded in plates and incubated with DHM or DHM combined with TGF-*β*1 for 18 h, the cells were labeled with EdU for 2 h at 37°C and 5% CO_2_, subjected to Apollo staining and Hoechst 33342 staining, and observed by fluorescence microscopy (Leica, DMi8, Germany).

### Seahorse XF Cell Mito Stress Test

The oxygen consumption rate (OCR) was detected with a Mito Stress test kit using a Seahorse XFe24 analyzer (Agilent Technologies). PHLFs were first seeded in XF24 cell culture plates at a density of 2×10^4^ cells/well and treated with DHM for 36 h. After the treatment was finished, the Cell Mito Stress assay medium was prepared according to the assay instructions and added to the XF24 plate. Finally, the OCR was detected with a Seahorse instrument.

### Histological Analysis

After inflation with 300 μL of 4% paraformaldehyde, the left lung of each mouse was removed and immersed in 4% paraformaldehyde for at least 24 h at room temperature. Then, the left lung was embedded in paraffin and sliced into 3 μM sections. The lung tissues were separately subjected to hematoxylin and eosin (H&E) staining, Sirius red staining, Masson staining, and immunohistochemistry. Assessment with the Ashcroft scoring system and immunohistochemistry were carried out as described previously (Wang Q. et al., 2021), and the severity of lung fibrosis was evaluated by the Ashcroft score (Ashcroft et al., 1988).

### Immunofluorescence Analysis

Each slide containing the lung tissue was incubated with anti-collagen I (Proteintech, 66761-1-Ig, 1:200) and anti-*α*-SMA (Proteintech, 14395-1-AP, 1:200) antibodies overnight at 4°C. After incubation, the slide was washed three times with PBS and then incubated with HRP- or 488-conjugated anti-mouse antibody and Alexa Fluor 594-conjugated anti-rabbit antibody (ZSGB-BIO, ZF-0512/0516, 1:400) for 1 h at room temperature. The slide was finally embedded with 4,6-diamino-2-phenylindole (DAPI) and analyzed under a fluorescence microscope (Wang Y. et al., 2021).

### Statistical Analysis

All experimental data were analyzed by using GraphPad Prism (San Diego, CA, United States). Other data are expressed as the mean ± standard error of the mean (SEM) or standard deviation (SD), and an independent Student’s t-test was administered to analyze the statistical significance of differences between two groups. The one-way analysis of variance (ANOVA), followed by Dunnett’s t post-hoc test or Tukey’s test, was also performed on raw data. If data were not normally distributed, the Kruskall–Wallis test was performed, followed by Dunn’s multiple comparison test. *p* < 0.05 was used to indicate statistical significance. All data were tested for normalization before analysis.

## Results

### *In vitro* Assessment of the Toxicity of Dihydromyricetin

**FIGURE 2:**
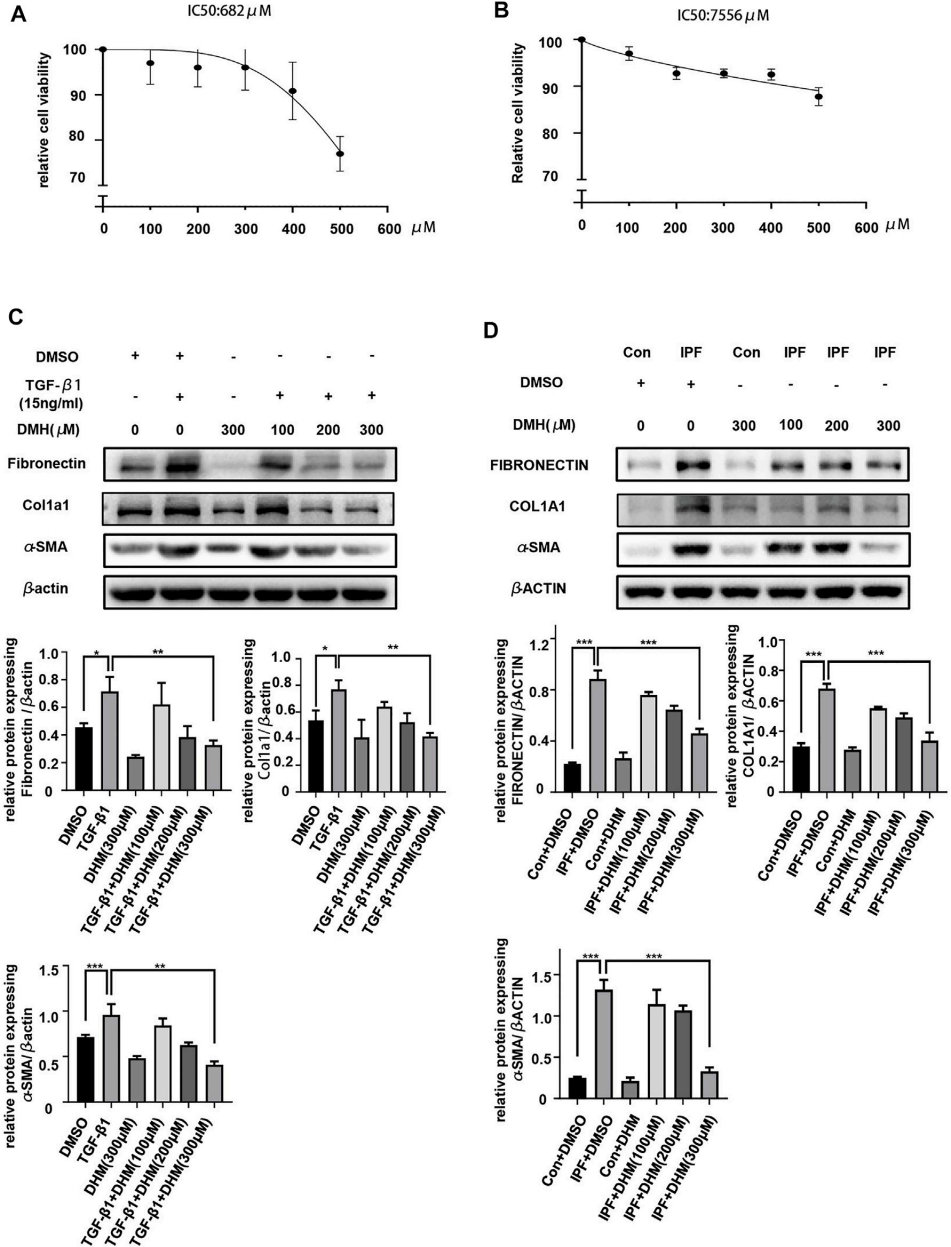
Effects of DHM on the differentiation of fibroblasts. **(A)** Toxicity detection of DHM on PHLFs by the CCK-8 assay (*n* = 6). **(B)** Toxicity detection of DHM on PMLFs by the CCK-8 assay (*n* = 4). **(C)** Western blotting analysis of fibronectin, Col1a1, and *α*-SMA in PMLFs treated with DHM (*n* = 5). **(D)** Western blotting analysis of fibronectin, COL1A1, and *α*-SMA expression in PHLFs treated with DHM (*n* = 5). The data are represented as the mean ± SD of three independent experiments. Ordinary one-way ANOVA analysis was applied. *, *p* < 0.05; **, *p* < 0.01; ***, *p* < 0.001.

To evaluate the toxicity of DHM *in vitro*, we treated PMLFs and PHLFs with increasing concentrations (100–500 μM) of DHM for 36 h. Notably, the CCK-8 assay revealed that the IC50 of DHM was 628 μM in PMLFs (Figure 2A) and 7556 μM in PHLFs (Figure 2B). These results also suggested that the population of cells was nearly unchanged when the concentration of DHM was below 400 μM. These results indicated that DHM may be safe for clinical applications.

### Dihydromyricetin Regulates the Differentiation of Fibroblasts to Myofibroblasts

The differentiation of fibroblasts to myofibroblasts is pivotal in the onset of pulmonary fibrosis, and myofibroblasts play important roles in the progression of pulmonary fibrosis (Peyser et al., 2019). Therefore, we evaluated the effects of DHM on the TGF-*β*1-stimulated differentiation of PMLFs and IPF-HLFs. The results of western blotting demonstrated that TGF-*β*1 stimulation significantly increased expression of the differentiation markers fibronectin, Col1a1, and *α*-SMA in PMLFs (Figure 2C). More interestingly, when compared to those in the N-HLFs, the levels of FIBRONECTIN, COL1A1, and *α*-SMA were much higher in the IPF-HLFs (Figure 2D). Moreover, DHM markedly suppressed the levels of these proteins in PMLFs stimulated with TGF-*β*1 and in IPF-HLFs. Furthermore, the degree to which differentiation was alleviated was greater in the group treated with DHM at a high concentration (300 μM) (Figures 2C,D). All these results revealed that DHM could not only attenuate the differentiation of fibroblasts to myofibroblasts induced by TGF-*β*1 but also lead to the dedifferentiation of IPF-HLFs, indicating that DHM may be effective for preventing pulmonary fibrosis and restoring the fibrotic lung tissue in clinical applications.

### Dihydromyricetin Facilitates Fibroblast Migration and Proliferation

**FIGURE 3:**
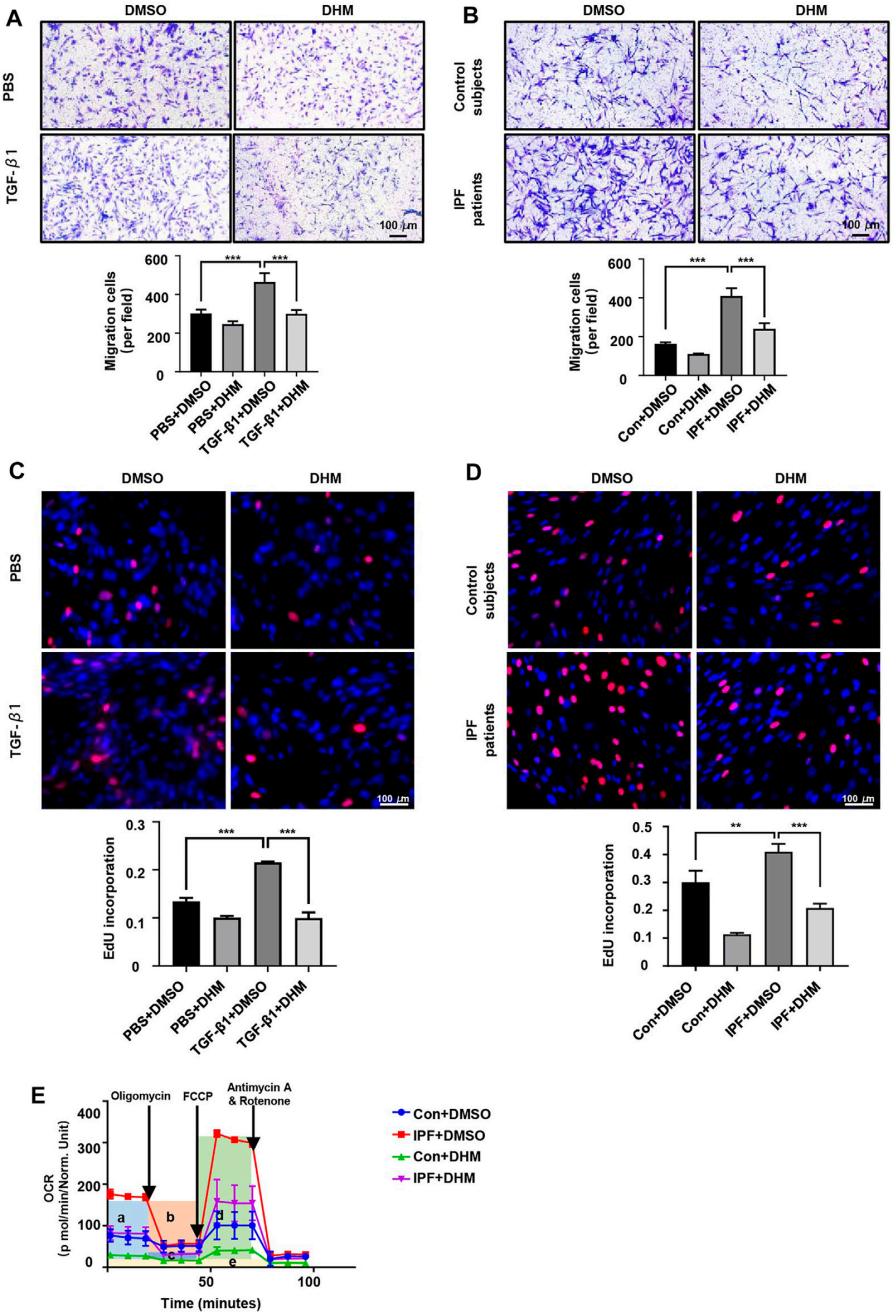
Effects of DHM on the proliferation, migration, and respiratory functions of fibroblasts. **(A)** Results for the Transwell assay in PMLFs treated with DHM. Images were captured at 100 × magnification (*n* = 5). **(B)** Results for the Transwell assay in PHLFs treated with DHM. Images were captured at 100 × magnification (*n* = 5). **(C)** Results for EdU staining in PMLFs treated with DHM. Images were captured at 200 × magnification (*n* = 5). **(D)** Results for EdU staining in PHLFs treated with DHM. Images were captured at 200 × magnification (*n* = 5). **(E)** Cell Mito Stress test results of PLHMs with DHM (a: basal respiration, b: ATP production, c: proton leak, d: maximal respiration, e: nonmitochondrial respiration) (*n* = 3). The data are represented as the mean ± SD of three independent experiments. Ordinary one-way ANOVA analysis was applied. *, *p* < 0.05; **, *p* < 0.01; ***, *p* < 0.001.

In addition to the functional effect of DHM on differentiation, its functional effects on migration and proliferation were assessed by the Transwell assay and EdU staining, respectively. The results of the Transwell assay revealed that the migration ability was much higher in myofibroblasts than in fibroblasts, but DHM at 300 μM remarkably suppressed the migration of PMLFs (Figure 3A) and PHLFs (Figure 3B) across the Transwell membrane. As shown by the EdU staining test, fewer EdU-positive cells were noted in the DHM treatment groups than in the untreated groups of PMLFs stimulated with TGF-*β*1 (Figure 3C). A similar result was also observed in the IPF-HLFs (Figure 3D). The proliferation function of the IPF-HLFs was much higher than that of the N-HLFs, and DHM remarkably suppressed the proliferation function of the IPF-HLFs.

### Dihydromyricetin Decreased the Increased Oxygen Consumption Rate in IPF-HLFs

Patients with IPF exhibit symptoms of dyspnea and need supplemental oxygen treatment, so the oxygen consumption of myofibroblasts in fibroblastic foci and those in healthy areas may differ. Thus, the OCR was detected by the Seahorse XF Cell Mito Stress test. As we hypothesized, the OCR (especially the basal respiration and maximal respiration stage) of the IPF-HLFs was much higher than that of the N-HLFs, and DHM significantly suppressed the basal and maximal respiration (Figure 3E) in both N-HLFs and IPF-HLFs. However, the suppression rate appeared to be higher in N-HLFs than IPF-HLFs.

### Dihydromyricetin Treatment Alleviated Pulmonary Fibrosis in a BLM Mouse Model

**FIGURE 4:**
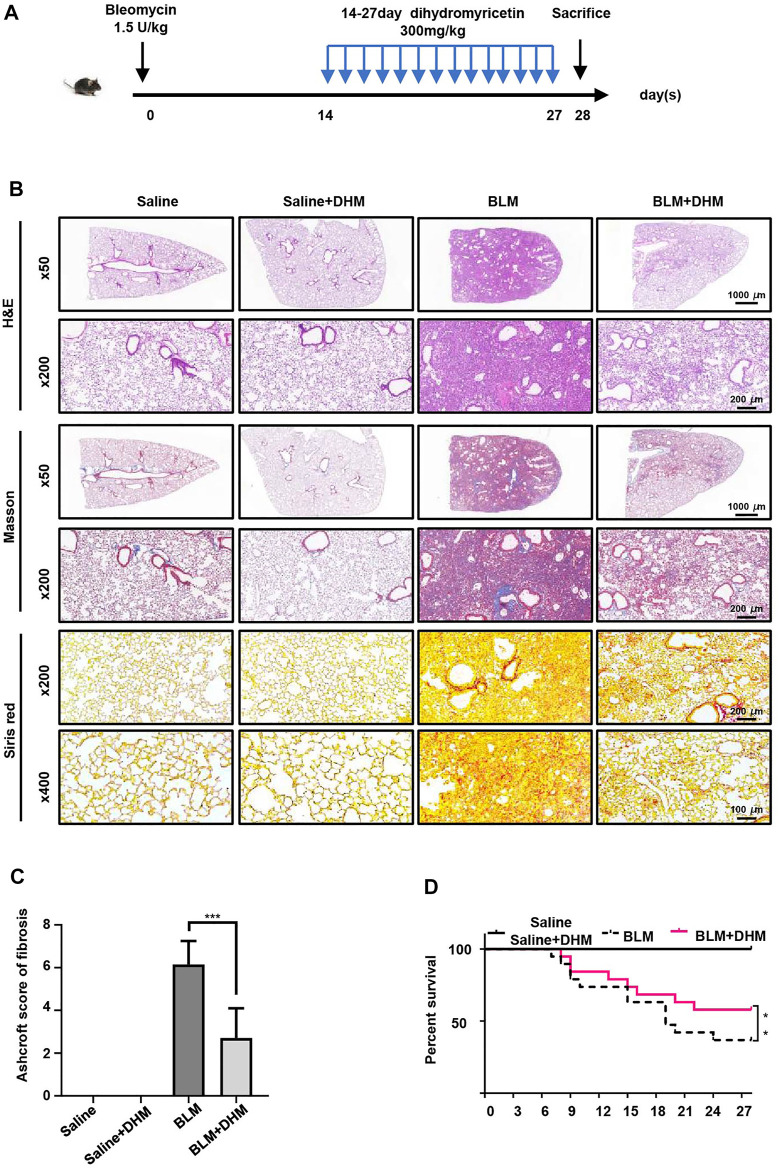
Effects of DHM on the severity of lung fibrosis in mice after BLM induction. Saline *n* = 6, DHM *n* = 6, BLM *n* = 6, BLM + DHM *n* = 6. **(A)** Time course of BLM and DHM administration. **(B)** Histological analysis of the severity of lung fibrosis in mice after BLM induction. Images were captured at 50 ×, 200 ×, and 400 × magnification. **(C)** Ashcroft score in different groups of mice (*n* = 6). **(D)** Survival ratio in different groups of mice. The data are represented as the mean ± SD. Two-sided Student’s t-test was applied. *, *p* < 0.05; **, *p* < 0.01; ***, *p* < 0.001.

DHM has been proven to suppress the TGF-*β*1-induced differentiation of fibroblasts to myofibroblasts and regulate the differentiation of IPF-HLFs *in vitro*. To assess the effects of DHM *in vivo*, we established BLM-induced pulmonary fibrosis in mice, treated the mice with DHM, and assessed the degree of pulmonary fibrosis (Figure 4A). Intratracheal injection of BLM induced the destruction of the normal lung architecture and the formation of lung fibrosis, but lesions in the lung tissue induced by BLM were alleviated in the DHM-treated group, as evidenced by H&E and Masson and Sirius Red staining (Figure 4B). Furthermore, the Ashcroft score of the DHM-treated group was lower than that of the untreated group (Figure 4C). Additionally, similar results were also observed by assessment of the survival curves, and the DHM-treated group had a higher survival rate than the BLM group (Figure 4D).

**FIGURE 5:**
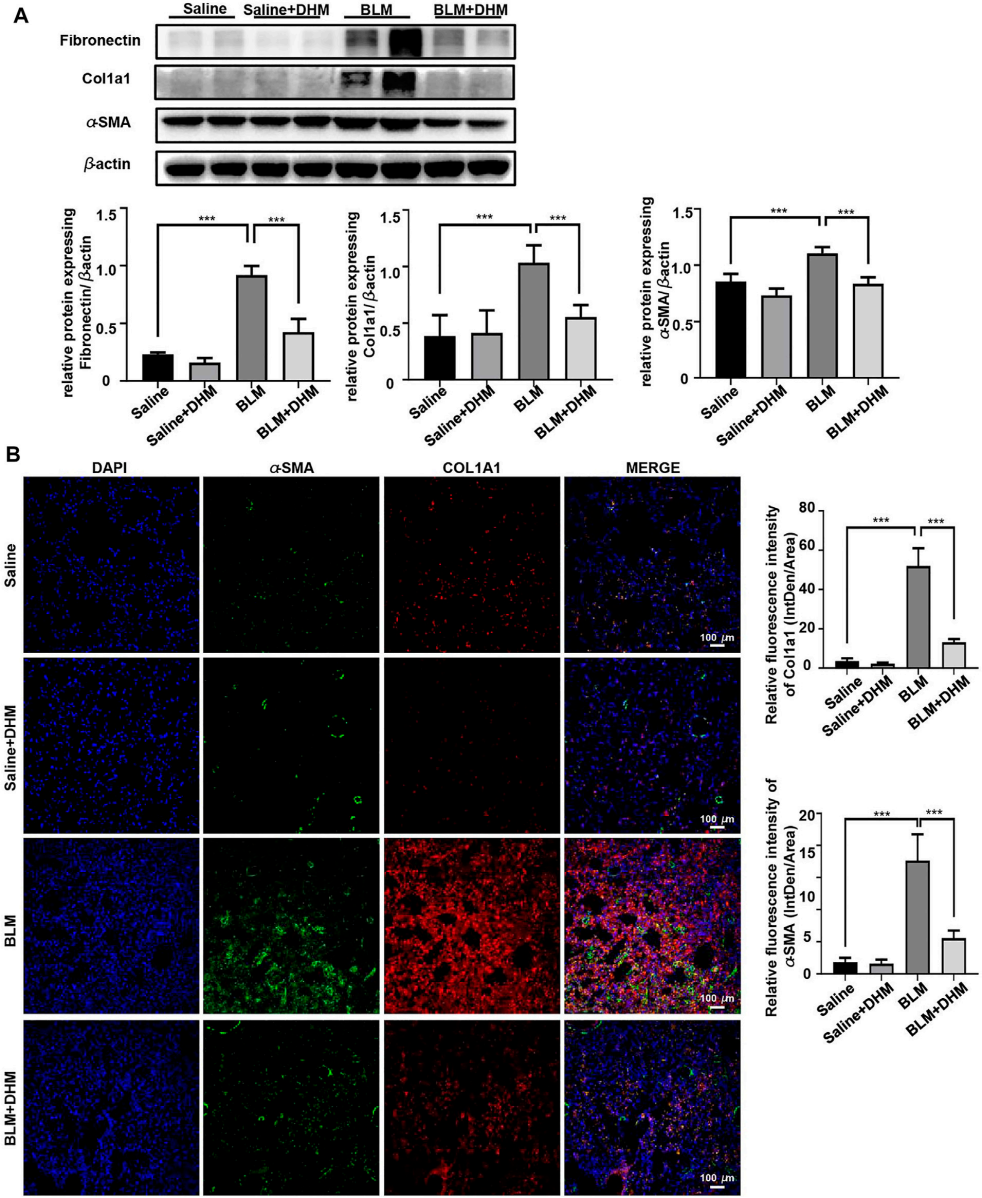
Effects of DHM on fibrosis markers in mice after BLM induction. Saline *n* = 6, DHM *n* = 6, BLM *n* = 6, BLM + DHM *n* = 6. **(A)** Western blotting analysis of fibronectin, Col1a1, and *α*-SMA expression in the lung homogenate from different groups. **(B)** Coimmunostaining of Col1a1 and *α*-SMA in the lung sections from different groups. Images were captured at 200 × magnification. The data are represented as the mean ± SD. Ordinary one-way ANOVA analysis was applied. *, *p* < 0.05; **, *p* < 0.01; ***, *p* < 0.001.

To further assess the effects of DHM on pulmonary fibrosis, we detected the levels of fibrotic markers (*α*-SMA, Col1a1, and fibronectin) by western blot analysis. Treatment with DHM markedly decreased the expression of fibrotic markers at the protein level in the BLM-induced mice (Figure 5A). Consistently, the same changes were also observed by immunohistochemistry (Supplementary Figure S1) and coimmunofluorescence (Figure 5B). The mice treated with BLM and DHM exhibited much lower levels of *α*-SMA, Col1a1, and fibronectin than those treated with BLM alone.

Although DHM was proven to be nontoxic *in vitro*, its toxic effects were also measured in mice. Excitingly, no obvious toxic effects of DHM on mice were observed, and no perceptible differences were found between the DHM group and the untreated group upon pathological staining of multiple organs, such as the heart, liver, spleen, and kidney (Supplementary Figure S2A), and the determination of biochemical liver and kidney indices, such as aspartate aminotransferase (AST), aminotransferase (ALT), urea (UR), and serum creatinine (CR) levels (Supplementary Figure S2B). All these findings indicate that DHM was well tolerated and safe for treating pulmonary fibrosis in a BLM-induced mouse model.

### Dihydromyricetin Regulates the STAT3/P-STAT3/GLUT1 Signaling Pathway

**FIGURE 6:**
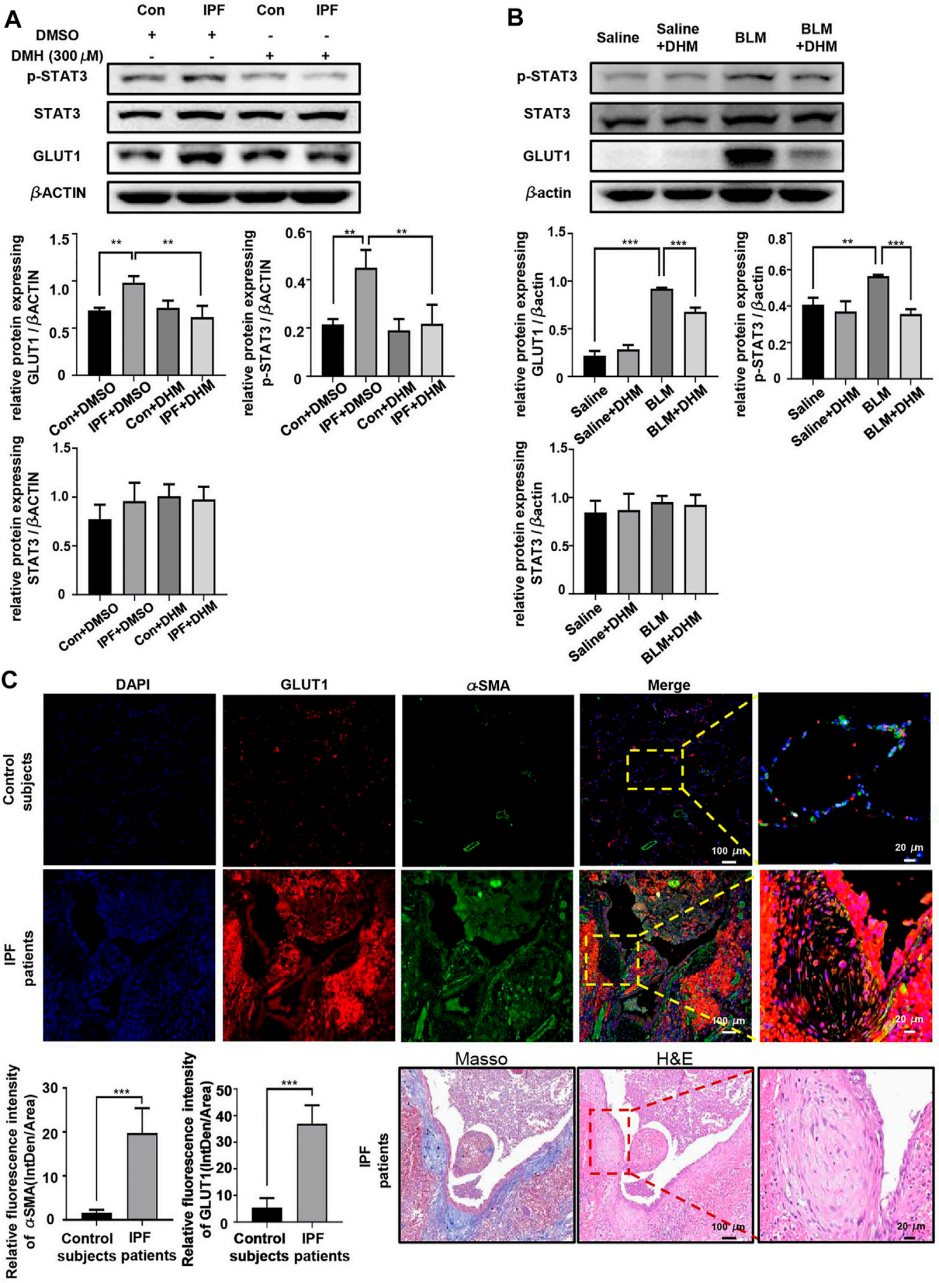
DHM affects the expression of pSTAT3/GLUT1. **(A)** Western blotting analysis of the levels of STAT3/p-STAT3/GLUT1 in PHLFs treated with DHM (*n* = 5). **(B)** Western blotting analysis of the levels of STAT3/p-STAT3/GLUT1 in the DHM-treated mouse model (*n* = 5). **(C)** Representative images for coimmunostaining of GLUT1 and *α*-SMA in the lung sections from control subjects and IPF patients. Images were captured at 100 × and 400 × magnification (control subjects = 5, IPF = 8). Representative images for Masson and H&E stain of an IPF patient. The data are represented as the mean ± SD. Ordinary one-way ANOVA analysis was applied. *, *p* < 0.05; **, *p* < 0.01; ***, *p* < 0.001.

Because DHM could significantly suppress the increased OCR in myofibroblasts, DHM might regulate the glucose metabolism. Glucose transporter 1 (GLUT1), the most widely distributed glucose transporter in mammalian cells (Rylaarsdam et al., 2019), is critical for glycolysis and has been reported to be regulated by the STAT3/p-STAT3 signaling pathway (Shang et al., 2020). Previous studies have also demonstrated that the STAT3/p-STAT3 signaling pathway participates in the progression of fibrosis and that DHM can regulate the STAT3/p-STAT3 pathway (Li et al., 2017; Celada et al., 2018). Therefore, we hypothesized that the antifibrotic function of DHM might be achieved by regulation of the STAT3/pSTAT3/GLUT1 signaling pathway. To verify this hypothesis, western blotting was used to analyze the levels of STAT3, pSTAT3, and GLUT1. As expected, compared to those in N-HLFs, the levels of pSTAT3 and GLUT1 in IPF-HLFs were much higher, and DHM suppressed the expression of pSTAT3 and GLUT1 *in vitro* (Figure 6A). Similar results were also found *in vivo*. We observed that BLM administration remarkably induced the expression of pSTAT3 and GLUT1 in mouse lung tissues, while DHM treatment could effectively block this pathway and suppress the expression of both proteins (Figure 6B). To further confirm our findings, we tested the expression of GLUT1 in the lung tissues of IPF patients and control subjects by coimmunofluorescence (Figure 6C). The level of GLUT1 was significantly increased in IPF patients, especially GLUT1 levels in the fibrotic foci of lung tissues.

**FIGURE 7:**
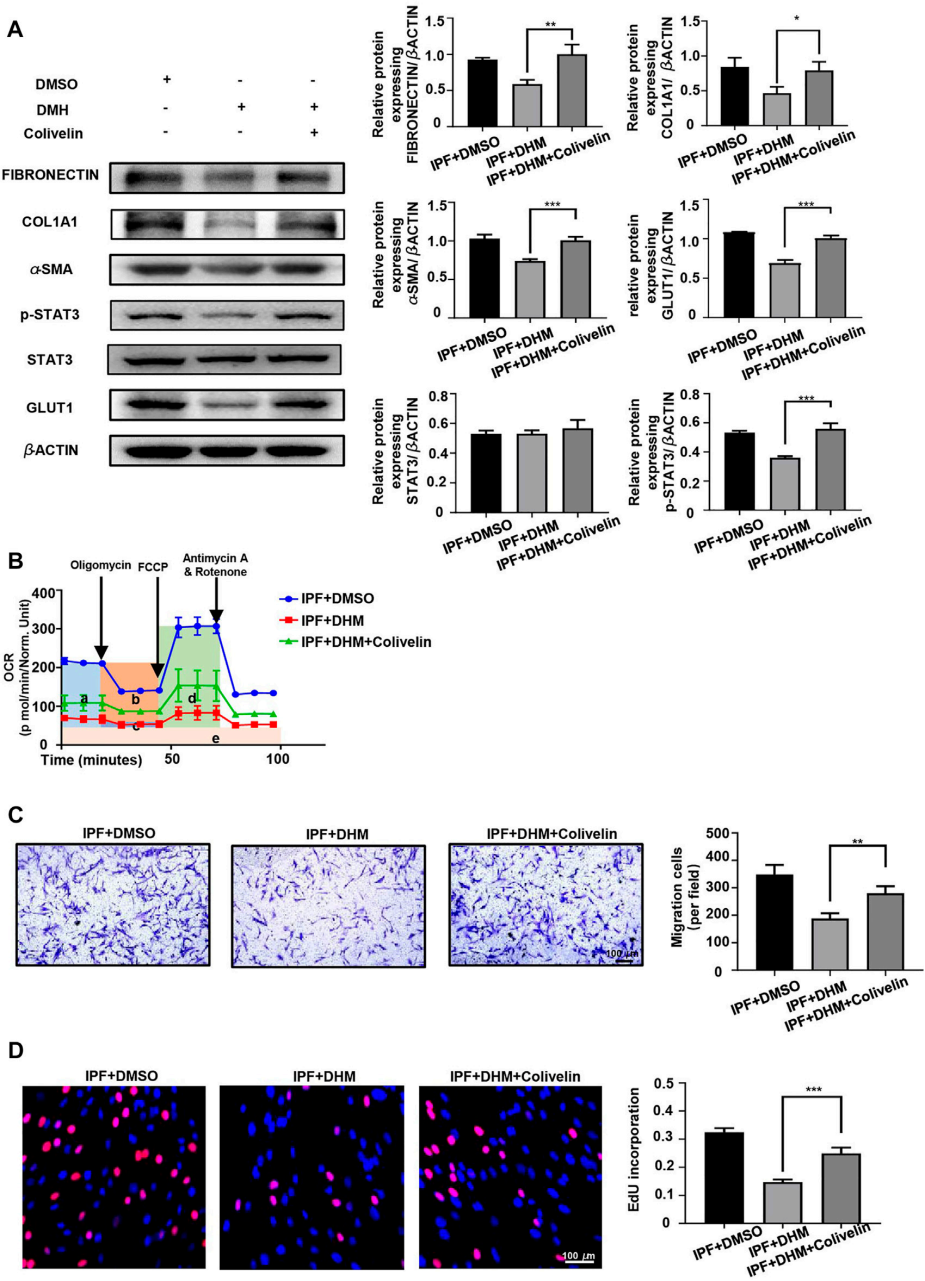
Colivelin reversed the effect of DHM on PHLFs. **(A)** Western blotting analysis of the levels of fibronectin, COL1A1, *α*-SMA, and STAT3/p-STAT3/GLUT1 in colivelin-treated PHLFs (*n* = 5). **(B)** Cell Mito Stress test results of colivelin-treated PLHMs (a: basal respiration, b: ATP production, c: proton leak, d: maximal respiration, e: nonmitochondrial respiration) (*n* = 3). **(C)** Results for the Transwell assay in colivelin-treated PLHMs (*n* = 5). **(D)** Results for EdU staining in colivelin-treated PLHMs (*n* = 5). The data are represented as the mean ± SD of three independent experiments. Two-sided Student’s t-test was applied. *, *p* < 0.05; **, *p* < 0.01; ***, *p* < 0.001.

Colivelin, a brain-penetrant neuroprotective peptide and potent activator of p-STAT3 that was reported to significantly increase p-STAT3 protein levels in BV-2 cells (Fang and Zhang, 2021), was used to test the effects of DHM on the STAT3/p-STAT3/GLUT1 axis. Western blot analysis revealed that colivelin not only reversed the DHM-induced suppression of p-STAT3 and GLUT1 expression in IPF-HLFs but also promoted the expression of fibrotic markers (Figure 7A). As colivelin increased the expression of GLUT1, the OCR (Figure 7B) was also increased after treatment with colivelin. Further studies on the functions of IPF-HLFs found that the DHM-induced suppression of migration (Figure 7C) and proliferation (Figure 7D) was also abrogated after treatment with colivelin.

## Discussion

**FIGURE 8:**
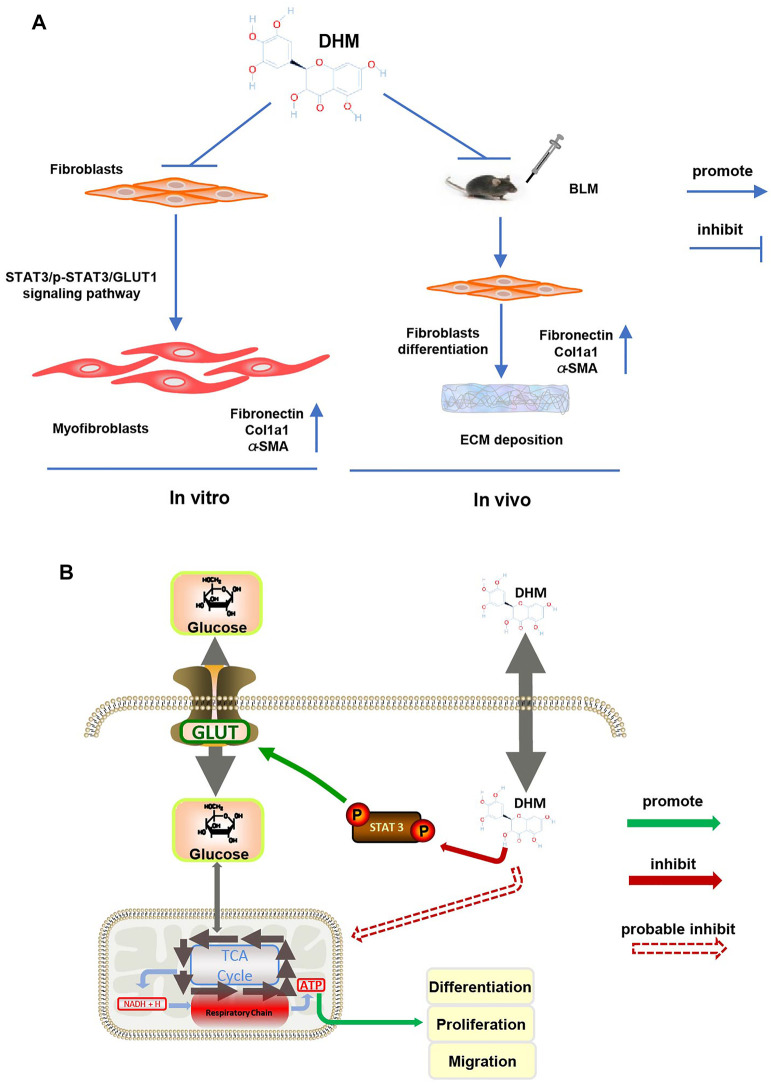
Mechanism of DHM on pulmonary fibrosis. **(A)** Schematic presentation of the mechanism in pulmonary fibrosis affected by DHM: TGF-*β*1 could induce fibroblasts to differentiate into myofibroblasts and overexpress fibronectin, Col1a1, and *α*-SMA *in vitro*, while DHM could suppress this differentiation progress by inhibiting the STAT3/p-STAT3/Glut1 signaling pathway; DHM could also alleviate pulmonary fibrosis induced by BLM *via* suppressing the differentiation of fibroblasts in the mouse model. **(B)** Intracellular signaling pathway of DHM: phosphorylation of STAT3 could promote the expression of GLUT1 and enhance the production of ATP, which eventually lead to the differentiation, proliferation, and migration of fibroblasts; DHM could regulate these functions by inhibiting the phosphorylation of STAT3. DHM might also regulate the respiratory chain directly and lead to the suppression of differentiation, proliferation, and migration of fibroblasts.

Our research showed that DHM could regulate the differentiation, migration, and proliferation functions of TGF-*β*1-stimulated fibroblasts and IPF-HLFs. DHM also alleviated BLM-induced pulmonary fibrosis in a mouse model (Figure 8A). Furthermore, regulation of the STAT3/p-STAT3/GLUT1 signaling pathway by DHM was found to be involved in this process (Figure 8B).

Before testing the effects of DHM on TGF-*β*1-induced fibroblasts and IPF-HLFs, we measured the toxicity of DHM in PMLFs and PHLFs. The CCK-8 assay was adopted to test the toxicity of DHM. Previous studies have suggested that the effective concentrations of DHM range from 1 to 200 μM *in vitro* and from 10 to 500 mg/kg/day *in vivo* (Zhang et al., 2018). Thus, in our research, five concentrations (100 μM, 200 μM, 300 μM, 400 μM, and 500 μM) were used. No obvious cell toxicity was observed at these concentrations. Interestingly, the toxicity of DHM was much lower in PHLFs than in PMLFs, suggesting that the activity in myofibroblasts might be greater than that in fibroblasts.

In recent studies on pulmonary fibrosis, TGF-*β*1 has been used to induce fibroblasts to differentiate into myofibroblasts. We also used 15 ng/ml TGF-*β*1 to stimulate PMLFs, and the concentration of DHM was set much lower than the IC50 value. Fibrotic markers (fibronectin, Col1a1, and *α*-SMA) were increased after TGF-*β*1 treatment, and these changes were suppressed by DHM in PMLFs. Although fibroblasts could be induced to differentiate into myofibroblasts by TGF-*β*1 *in vitro*, they did not completely imitate the functions of pulmonary myofibroblasts *in vivo* (Epstein et al., 2020). Then, we isolated IPF-HLFs and N-HLFs. Interestingly, the levels of fibrosis-associated proteins were much higher in IPF-HLFs than in N-HLFs, even without stimulation with TGF-*β*1. Treatment with DHM not only suppressed the expression of these proteins in PMLFs but also seemed to demonstrate stronger inhibitory effects in IPF-HLFs. These findings provide firm evidence that DHM did in fact regulate the differentiation of IPF-HLFs. In addition to differentiation, the abnormally elevated migration and proliferation of myofibroblasts are also considered important in the progression of pulmonary fibrosis (Wollin et al., 2015). In our research, we found that the proliferation and migration functions of IPF-HLFs were also much stronger than those of N-HLFs. DHM was also found to affect these functions. The increased migration and proliferation of TGF-*β*1-induced fibroblasts or IPF-HLFs were clearly attenuated with DHM treatment. Our findings obtained with PMLFs and PHLFs indicated that DHM could not only suppress the activation effects of TGF-*β*1 on fibroblasts but also regulate the functions of IPF-HLFs. All these results suggest DHM might be a multifunctional medicine for the prevention and treatment of pulmonary fibrosis.

Shortness of breath and increasing cough are the most common clinical symptoms of IPF in patients (Pleasants and Tighe, 2019). Both damage to the normal lung tissue and oxygen exchange dysfunction contribute to hypoxia and respiratory failure in these patients (Richeldi et al., 2017). Even though under hypoxic conditions, myofibroblasts in the lung tissue still maintain a highly proliferative state (Senavirathna et al., 2018), we wondered whether the abnormal metabolism is related to increased proliferation. As the main nutrient used in eukaryotic cell proliferation is glucose and the metabolism of glucose mainly occurs in the mitochondria, we used the Seahorse XF Cell Mito Stress test to detect the OCR in fibroblasts and myofibroblasts and the effects of DHM on the OCRs. As illustrated by the OCR results, basal respiration, ATP production, and maximal respiration were all increased in the IPF-HLFs compared with the N-HLFs. More importantly, DHM significantly attenuated these functions in both N-HLFs and IPF-HLFs. As previously mentioned, the N-HLFs were more suppressed than IPF-HLFs by DHM in the suppression rate, and this phenomenon might be caused by the antagonism between the antioxidative effects of DHM and the high expression of GLUT1 on IPF-HLFs. DHM might suppress the mitochondrial respiration directly on N-HLFs and IPF-HLFs (Figure 8B). However, on IPF-HLFs, the high expression of GLUT1 contributed to the increase of OCRs and weakened the suppression effects of DHM. This finding also suggested that GLUT1 might participate into the progression of pulmonary fibrosis by regulating mitochondrial respiratory.

After verifying the effects of DHM on TGF-*β*1-induced PMLFs and IPF-HLFs, we also detected the effects of DHM on BLM-induced pulmonary fibrosis in a mouse model. According to previous studies, DHM can suppress liver fibrosis and renal interstitial fibrosis by inhibiting inflammation and injury induced by TGF-*β*1. To determine the therapeutic effects of DHM on pulmonary fibrosis, DHM treatment was applied after the formation of pulmonary fibrosis in our experiment. As the expression of fibrogenic genes started increasing on the 7th day and peaked on the 14th day after intratracheal injection of BLM, DHM treatment was started at the fibrosis stage from the 14th day to the 27th day following BLM induction. The mice were sacrificed on the 28th day. To our delight, the mice in the BLM + DHM group demonstrated greater remission of pulmonary fibrosis than those in the BLM group. Notably, no differences in survival rates were observed between the BLM group and the treatment group over the first 14 days (the inflammation stage), but in the fibrosis stage, the treatment group exhibited higher survival rates than the BLM group. These results suggest that DHM treatment after the formation of fibrosis effectively alleviated pulmonary fibrosis and was well tolerated in the BLM-induced mouse model.

As found previously, an abnormal metabolism was present in IPF-HLFs, and the abnormal metabolism can drive myofibroblasts to proliferate and secrete ECM in excess, ultimately aggravating pulmonary fibrosis. This process is similar to the process by which growth and migration are driven by the aberrant metabolism in tumors (Park et al., 2020). Studies have found that the STAT3/p-STAT3/GLUT1 pathway is involved in the metabolism and proliferation of cancer cells (Xu et al., 2016; Nagarajan et al., 2017). In our study, DHM downregulated the levels of p-STAT3 and GLUT1 in myofibroblasts. Further investigation revealed that DHM also suppressed the increase in p-STAT3 and GLTU1 levels induced by BLM in the model mice. Many studies have demonstrated that TGF-*β*1 can promote fibrosis *via* the STAT3/p-STAT3 pathway (Chakraborty et al., 2017; Dees et al., 2020); however, few of these studies have explored the effects of abnormal STAT3/p-STAT3 expression on IPF-HLFs. Our research showed that even without TGF-*β*1 stimulation, the increase in p-STAT3 still aggravated the dysfunction of IPF-HLFs, and regulating the level of p-STAT3 modulated the functions of IPF-HLFs. Some research has indicated that upregulated expression of GLUT1 in fibroblasts and macrophages can exacerbate pulmonary fibrosis (El-Chemaly et al., 2013; Andrianifahanana et al., 2016), and the pathways in which GLUT1 is involved are mostly focused on inflammation. Hence, this might be the first study to show that GLUT1 is regulated by p-STAT3 in IPF-HLFs and that this regulation participates in the abnormal glucose metabolism. Coimmunofluorescence analysis of lung tissues from IPF patients illustrated that the expression of GLUT1 in myofibroblasts located in fibroblastic foci was remarkably increased; more interestingly, the blood supply around the fibroblastic foci was also remarkably increased. These abnormal lung tissue features also explain why even though IPF patients always suffer from hypoxia, the activity of myofibroblasts in their lung tissue remains at a high level and promotes the progression of fibrosis. To further confirm the regulatory effects of DHM on the STAT3/p-STAT3/GLUT1 pathway, colivelin was used to increase the expression of p-STAT3. As expected, colivelin specifically upregulated the expression of p-STAT3, and the expression of GLUT1 was subsequently increased. To our delight, the DHM-induced suppression of myofibroblast differentiation, proliferation, and migration was nearly reversed by colivelin in DHM and colivelin-treated cells. However, the suppressed respiration functions of IPF-HLFs by DHM were partially reversed by colivelin, and this might be related to the antioxidative effects of DHM and the direct effects of DHM on mitochondrial respiration. These findings suggest that the antifibrotic effects of DHM might mainly depend on the STAT3/p-STAT3/GLUT1 signaling pathway.

Although pirfenidone and nintedanib have been approved for the treatment of IPF, the outcomes of IPF patients are still not optimistic, and new treatments are urgently needed (Caminati et al., 2019). As traditional Chinese medicine has attracted increasing attention, studies have illustrated that some herbal extracts have antifibrotic effects by suppressing the inflammation and oxidative stress induced by cytokines such as IL-6 and TGF-*β*1 (Wang et al., 2020; Wang Z. et al., 2021), but few of these medicines have been proven effective in clinical IPF patients. In our study, we isolated IPF-HLFs from the lung tissues of IPF patients, and DHM effectively regulated the differentiation, migration, and proliferation of IPF-HLFs. This finding provides substantial evidence that DHM is a candidate medicinal treatment for IPF. More interestingly, our findings suggest that GLUT1 participates in the differentiation of fibroblasts in the development of pulmonary fibrosis. Similar changes were also found in cancer cells (Ancey et al., 2018) as GLUT1 was found to promote the proliferation and migration of cancer cells (Oh et al., 2017; Gonzalez-Menendez et al., 2018; Li et al., 2020) and the regulation of GLUT1 and the glucose metabolism could suppress the proliferation and migration of cancer cells (Liu et al., 2012; Zambrano et al., 2019; Heydarzadeh et al., 2020). All these findings have led to new targeted therapy for cancer treatment. Our findings suggest that DHM could alleviate pulmonary fibrosis by suppressing GLUT1 in mice and might also lead to a breakthrough in the treatment of IPF by regulating the glucose metabolism in fibroblasts.

Our study also has some limitations. Although no perceptible toxicities were detected *in vivo* or *in vitro* in our study, the blood concentration and pharmacokinetics of DHM remain unclear and should be investigated in the future. Second, DHM is a lipid-soluble drug, which limits the bioavailability of DHM. Thus, more research to improve the bioavailability of DHM is needed.

## Conclusion

Our research suggests that DHM could alleviate pulmonary fibrosis *in vitro* and *in vivo via* the STAT3/p-STAT3/GLUT1 signaling pathway. The antifibrotic effects of DHM are achieved by regulating the abnormal glucose metabolism in myofibroblasts. This finding provides us with new insight indicating that metabolic therapy might be a breakthrough for IPF treatment. We would be delighted if our work could act as a sufficient theoretical basis for this new research direction.

## Data Availability

The original contributions presented in the study are included in the article/Supplementary Material, further inquiries can be directed to the corresponding authors.

## Supplementary Material

Supplementary Figure S1 Immunohistochemistry of fibronectin, Col1a1, and *α*-SMA in the lung sections from different groups (*n* = 6).

Supplementary Figure S2 Toxic effects of DHM on mice. **(A)** Histological analysis of the inflammation levels of multiorgans in different groups (*n* = 6). Images were captured at 100 × magnification. **(B)** Levels of biomarkers of the liver and kidney in different groups (*n* = 6). The data are represented as the mean ± SD. Two-sided Student’s t-test was applied.
